# Tuberculosis-related stigma and its determinants in Dalian, Northeast China: a cross-sectional study

**DOI:** 10.1186/s12889-020-10055-2

**Published:** 2021-01-04

**Authors:** Xu Chen, Liang Du, Ruiheng Wu, Jia Xu, Haoqiang Ji, Yu Zhang, Xuexue Zhu, Ling Zhou

**Affiliations:** School of Public Health, Dalian Medical University, 9 Western Section, Lvshun South Street, Lvshunkou District, Dalian, People’s Republic of China 116044

**Keywords:** Tuberculosis, Stigma, Associated factors, China

## Abstract

**Background:**

The stigma of tuberculosis (TB) poses a significant challenge to TB control because it leads to delayed diagnosis and non-adherence. However, few studies on TB-related stigma have been completed in China. The aim of the current study was to explore the status of TB-related stigma and its associated predictive factors among TB patients in Dalian, Northeast China.

**Methods:**

An institution-based, cross-sectional survey was conducted among outpatients at Dalian Tuberculosis Hospital in Liaoning Province, Northeast China. Data were collected by using a questionnaire that measured TB-related stigma, treatment status, anxiety, social support, doctor-patient communication and so on. A multiple linear regression model was used to determine the predictors of TB-related stigma.

**Results:**

A total of 601 eligible participants were recruited. The mean score for TB-related stigma was 9.07, and the median score was 10. The average scores for anxiety, social support and doctor-patient communication were 4.03, 25.41 and 17.17, respectively. Multiple linear regression analysis revealed that patients who were female (*β* = 1.19, 95% *CI*: 0.38–2.01, *P* < 0.05), had self-assessed moderate or severe disease (*β* = 1.08, 95% *CI*: 0.12–2.03 and *β* = 1.36, 95% *CI*: 0.03–2.70, respectively, *P* < 0.05), and had anxiety (*β* = 0.38, 95% *CI*: 0.30–0.46, *P* < 0.001) were more likely to have a greater level of TB-related stigma than their counterparts. However, a significantly lower level of TB-related stigma was observed in patients with good social support (*β* = − 0.25, 95% *CI*: − 0.33--0.17, *P* < 0.001) and doctor-patient communication (*β* = − 0.14, 95% *CI*: − 0.29--0.00, *P* < 0.05).

**Conclusions:**

This study showed that stigma among TB patients was high. Targeted attention should be paid to female patients and patients with moderate or severe disease in TB stigma-related interventions. Moreover, the important role of social support and doctor-patient communication in reducing TB-related stigma should also be emphasized.

**Supplementary Information:**

The online version contains supplementary material available at 10.1186/s12889-020-10055-2.

## Background

Tuberculosis (TB) is a chronic infectious disease that severely affects the health of millions of people each year and is a major public health problem worldwide [1]. Like those with human immunodeficiency virus (HIV)/acquired immune deficiency syndrome (AIDS) and leprosy, TB patients also face deep-rooted and persistent stigma [2–4]. This can be felt in different social environments, such as the home, workplace, and community, resulting in serious impacts on TB patients [5, 6]. TB-related stigma has become a formidable challenge for TB prevention and control [7, 8]. However, there is also a growing awareness of the need to address the stigma related to TB, a major social problem [9, 10].

Stigma was defined by Goffman as “an attribute that is deeply discrediting” that demeans people “from a whole and usual person to a tainted, discounted one” [11]. Studies in different contexts have reported that approximately 42–82% of TB patients suffer from stigma, while studies from China show a 45.32% prevalence of stigma [9, 12, 13]. TB-related stigma is generally the product of the exaggerated concept of contagiousness and fear of infection [12, 14, 15]. Due to a lack of understanding, people have false perceptions that TB is incurable and is highly contagious throughout treatment [7, 16]. Sometimes inappropriate information in the media, such as fragmented or improperly presented information, results in the social reinforcement of TB-related stigma [17]. In addition, TB and HIV/AIDS share common physical signs and symptoms, such as weight loss, which also often leads to its association with HIV/AIDS [12, 18]. Importantly, HIV/AIDS is a risk factor for the development of TB, this could be one of the leading drivers behind the misconception about everyone with TB is HIV/AIDS positive [1, 19]. The negative characteristics of unethical or promiscuous sex that have been associated with HIV/AIDS, as well as its deadly nature, are also assumed in TB patients [3, 7, 16]. The complex relationship between TB and HIV/AIDS stigmatizes TB patients [2, 8, 20].

TB-related stigma has been identified as a major obstacle to patients seeking medical care and completing a full course of treatment [5, 21, 22]. A study found that patients who have a higher level of stigma are 5.88 times more likely to present sputum delays than patients who have a lower level of stigma [23]. Some 3 million TB patients worldwide, a daunting number, went unreported in 2018 alone [1]. Stigma also leads to decreased self-esteem and a poor quality of life in patients, hinders symptoms of infection or disease from being disclosed, and undermines TB screening efforts in the home and workplace [20, 24, 25]. It also causes patients to avoid contact and interaction with others and to isolate themselves in response to social attitudes and behaviours, which may have an impact on their mental and physical health [26, 27]. Studies have also shown that patients who have stigma are 11 times more likely to be depressed, and those who have high levels of stigma are more likely to have severe depression than those who do not [24, 28]. It is important, therefore, to develop interventions to effectively reduce stigma through research into the factors associated with stigma in TB patients.

A small number of studies have been conducted to identify factors associated with TB-related stigma. A study conducted in India showed that some sociodemographic characteristics, such as caste, number of family members, and place of residence, were important factors influencing stigma among TB patients [23]. In a national survey related to stigma in Ethiopia, stigma was found to be associated with educational status, poverty and lack of awareness of TB [8]. A cross-sectional study conducted in Wolaita Sodo showed that patients who were in the intensification phase; were co-infected with HIV; had poor social support; and used substances such as alcohol, khat and cigarettes were more likely to experience perceived TB stigma [15]. Studies on stigma among TB patients in China have also been performed. A study completed in Hubei Province, Central China, claimed that knowledge about TB, family functioning, and doctor-patient communication were negatively correlated with TB-related stigma [29].

Despite some progress in TB control in recent years, the burden of TB in China remains high, second only to India, accounting for 9% of the global total [1]. As far as we know, there have been no studies on TB stigma conducted in Northeast China. We also observed that studies in other parts of China explored factors associated with stigma in terms of only sociodemographic characteristics [28], drug compliance and quality of life [30], TB knowledge and family functioning [29]; there is a lack of research on associated treatment status and anxiety. Moreover, the factors associated with TB-related stigma may vary depending on social and cultural backgrounds and region [16]. Therefore, we conducted a cross-sectional study in Dalian, Liaoning Province, Northeast China. The purpose was to assess the status of TB-related stigma among TB patients in Dalian, Northeast China, and to analyse factors associated with TB-related stigma in terms of sociodemographic characteristics, treatment status, substance use, anxiety, social support and doctor-patient communication.

## Methods

### Study area

The study was conducted in Dalian, Liaoning Province, Northeast China. Liaoning Province is a coastal province with a strong economy in Northeast China. In 2018, the legally required reported incidence of TB was 55.81/100,000, and the mortality rate was 0.49/100,000 in Liaoning Province [31]. These rates are lower than those in Heilongjiang Province and higher than those in Jilin Province. The incidence rate ranks 15th among the 31 regions in China and is lower than the national incidence rate of 59.27/100,000. The death rate ranks 4th in China and is higher than the national death rate of 0.23/100,000 [31]. Dalian city is an economically developed area in Liaoning Province. Dalian city is located in the southern part of Liaoning Province, bordering the Yellow Sea in the east and the Bohai Sea in the west. It covers an area of 12,574 km^2^. The jurisdiction comprises 7 municipal districts, 1 county and 2 county-level cities. In 2018, there were 5.952 million registered residents and 6,987,500 permanent residents. Based on the permanent resident population, the per capita gross domestic product (GDP) was 109,644 yuan. In 2016, the incidence of TB in the general population of Dalian was 64.66/100,000 [32]. Dalian Tuberculosis Hospital is the only specialized hospital for TB control in Dalian. It is divided into north and south parts located in Dalian Ganjingzi district and Pulandian district, respectively. The hospital is responsible for the diagnosis, treatment and prevention of TB in Dalian.

### Study design and participants

An institution-based, cross-sectional survey was conducted between September 2019 and January 2020 at the Dalian Tuberculosis Hospital. With the help of hospital staff, outpatients who were on a waiting list and met the inclusion criteria were recruited. The inclusion criteria for patients included the following: (1) patients who had a diagnosis of TB with no mental illness, (2) patients aged greater than or equal to 18 years, (3) patients with clear consciousness, clear communication, and the ability to understand the contents of the questionnaire, and (4) patients who agreed to participate in this study and could honestly express their views on the problem. Patients who participated in the study independently answered questionnaires administered by the investigators, and patients with limited literacy completed the questionnaires with the assistance of the investigators. A total of 606 patients were investigated in this study. Due to time constraints, 5 patients were excluded from the study because they did not complete the investigation. Therefore, 601 eligible patients were included in this study, and the participation rate was 99.17%. To ensure the quality of the collected questionnaire data, an investigation team consisting of 7 graduate students completed a training class. The training content mainly included the research purpose and content, the implementation of the field investigation, the screening of subjects, the general requirements of the investigation and matters needing attention. After the training, the surveyors were assessed on the proficiency level of the questionnaires, matters needing attention in the survey and other contents. After approving the assessment, questionnaires were administered over a 5-month period. After each questionnaire was completed, it was placed in a sealed envelope and held by the principal researchers to ensure the confidentiality of the data.

### Data collection

The data were collected through structured questionnaires designed by the researchers. The questionnaire was designed on the basis of a large amount of relevant literature and consultations with experts in related fields. To ensure that the respondents accurately understood the meaning of each item in the questionnaire, we conducted a presurvey at the study site and modified the questionnaire accordingly. The questionnaire was composed of six parts: sociodemographic characteristics, TB-related stigma, statuses of treatment and substance use, anxiety, social support, and doctor-patient communication. Sociodemographic characteristics included sex, age, education, marital status, employment status and family size (number of people living together). Treatment status mainly focused on TB treatment history and hospitalization history, comorbidities, self-assessed disease severity, self-assessed disease cure confidence and self-assessed physical health status; substance use mainly related to the use of alcohol and cigarettes (See Additional file 1).

Stigma refers to the experience of inner shame caused by the disease, which is the embodiment of the complex psychosocial response of the patient [33, 34]. Stigma can be divided into three main categories: experienced stigma (experiences of exclusion and/or discrimination), anticipated stigma (perception, expectation and/or fear of stigma) and internalized stigma (loss of self-esteem, loss of dignity, fear and/or shame) [7]. In this study, these classifications were applied, and TB-related stigma was measured from three dimensions: negative experience, emotional response and coping style. The TB-related stigma scale was developed in combination with the social and cultural characteristics of China and consists of 9 items [34]. Negative experiences were measured by four items comprising the attitudes and behaviours of family, neighbours and friends. Emotional responses were assessed by two items concerning self-esteem and shame. Coping styles were measured by three items including fear, isolation and disclosure of TB status. Each item on the scale is rated on a 4-point Likert scale ranging from 0 (strongly disagree) to 3 (strongly agree). The scores for each item are added up to obtain a total score, ranging from 0 to 27. Higher scores indicate higher levels of stigma in TB patients. Scores above 9 are considered to indicate moderate or high levels of stigma [35]. Patients who reported stigma were classified according to the mean and standard deviation (SD). The scale had good validity and internal consistency [36], and the Cronbach’s α was 0.90 in this study.

Anxiety in TB patients was measured using the self-rated, 7-item Generalized Anxiety Disorder scale (GAD-7) [37]. The scale had excellent internal consistency in the current study (Cronbach’s α =0.95). The scale collects data about the frequency of symptoms described in each item over the past 2 weeks. Each item has four answer options: 0 (not at all), 1 (several days), 2 (more than half of the days), and 3 (nearly every day). The total score ranges from 0 to 21, with higher scores reflecting more severe anxiety. In previous studies, anxiety was divided into four levels with 5, 10, and 15 as cut off points [37].

The Social Support Rating Scale (SSRS), which is widely used in China and has shown good reliability and validity, was used to assess the status of patients’ social support [38, 39]. The Cronbach’s α of this scale was 0.91 in the current study. The SSRS scale consists of 10 items in three dimensions: objective support, subjective support and utilization of social support. Scores range from 12 to 66 and are used to assess an individual’s overall social support. Higher overall scores indicate better social support.

Doctor-patient communication was measured by four questions involving satisfaction with doctors’ interpretations of the condition, interpretation of anti-TB drug usage and dosage, interpretation of adverse drug reactions, and service attitude [29]. Participants were asked to rate the questions on a scale of 1 (very dissatisfied) to 5 (very satisfied). Doctor-patient communication scores range from a minimum of 4 to a maximum of 20. The higher the doctor-patient communication score, the better the doctor-patient communication is. The four items for measuring doctor-patient communication had acceptable internal consistency in our study (Cronbach’s α =0.71).

### Statistical analysis

After the questionnaire, which was collected on-site, was checked for completeness and correctness, the original data were entered into a database in EpiData 3.1 (EpiData Association, Odense, Denmark) software by means of double entry, and consistency was assessed. All data analyses were performed by using IBM SPSS statistics version 21.0 (IBM Corp, Armonk, NY). We analysed the means and SDs or medians and interquartile ranges for continuous data, and the frequencies and percentages for categorical data. The rank sum test was used to compare TB stigma scores among different groups. Mann-Whitney U test was used for comparison between the two groups, and Kruskal-Wallis H test was used for comparison between multiple groups. Spearman’s correlation analysis was used to assess correlations between anxiety, social support, doctor-patient communication and TB-related stigma. Variables that were statistically significant in the univariate analyses were entered into multiple linear regression analysis to correct for confounders and identify factors associated with TB-related stigma. All comparisons were two-tailed, and *P* < 0.05 was considered statistically significant.

## Results

### Sociodemographic characteristics and their relationships with TB-related stigma

The 601 participants who participated in the study ranged in age from 18 to 88 years, with a mean (SD) age of 45.16 (17.51) years; approximately half (49.92%) of the participants were 45 years or older. Nearly two-thirds of the participants (62.56%) were male, and more than half of the participants (57.40%) had a high school education or above. Most of the participants (72.38%) were married, while only 27 (4.49%) were divorced or widowed. Approximately one-third of the participants (32.78%) were unemployed, and nearly three-quarters (73.21%) reported a family size of three people or more. Of the total participants, the mean (SD) score of TB-related stigma was 9.07 (5.62), and the median score was 10. The average scores for each item in the dimensions of negative experience, emotional response, and coping style were 0.87, 1.10, and 1.13, respectively. More than half of the patients (50.42%) scored more than 9, and 19.47% scored more than 14. Approximately half of the patients (48.42%) were reluctant to tell their friends or colleagues that they had TB, and 50.75% avoided friends and neighbours because of their TB status. The univariate analysis showed that the TB-related stigma scores were statistically significant among different sexes, employment statuses, and family sizes (*P* < 0.05) (Table 1).

**Table 1:** Sociodemographic characteristics of the patients and their relationships with TB-related stigma

Variables	Patients		TB-related stigma		*P*
	n	%	median	interquartile range	
Sex					**0.014**
Male	376	62.56	9.00	3.00–12.00	
Female	225	37.44	10.00	6.00–14.00	
Age					0.323
≤ 24	77	12.81	9.00	2.00–13.00	
25~	224	37.27	10.00	5.00–13.00	
45~	200	33.28	9.00	4.00–14.00	
≥ 65	100	16.64	10.00	5.25–13.75	
Educational status					0.078
Primary or less	75	12.48	10.00	5.00–15.00	
Secondary	181	30.12	9.00	3.00–13.00	
High school or above	345	57.40	10.00	5.00–13.00	
Marital status					0.171
Unmarried	139	23.13	9.00	6.00–12.00	
Married	435	72.38	10.00	4.00–13.00	
Divorced or widowed	27	4.49	11.00	6.00–17.00	
Unemployment					**0.001**
Yes	197	32.78	10.00	6.00–15.00	
No	404	67.22	9.00	3.00–12.00	
Family size					**0.009**
1	38	6.32	10.00	7.00–15.00	
2	123	20.47	10.00	7.00–15.00	
≥ 3	440	73.21	9.00	3.00–12.00	

### Treatment and substance use statuses and their relationships with TB-related stigma

Of the respondents, the number of patients with newly diagnosed TB (80.03%) was approximately four times higher than the number of those with relapse TB. Similarly, the number of TB patients with a history of hospitalization (80.87%) was approximately four times higher than the number of those without a history of hospitalization. More than a quarter of the participants (27.12%) said they also had other medical conditions, and 371 (61.73%) felt their condition was mild. A large percentage of participants (73.54%) said they were very confident in achieving TB cure, and more than half (54.24%) reported that their physical health was moderate or poor. Among the participants, 142 (23.63%) smoked or consumed alcohol. The univariate analyses indicated that there were statistically significant differences in TB-related stigma scores according to self-assessed disease severity, self-assessed TB cure confidence, and self-assessed physical health status (*P* < 0.05) (Table 2).

**Table 2:** Treatment and substance use statuses and their relationships with TB-related stigma

Variables	Patients		TB-related stigma		*P*
	n	%	median	interquartile range	
TB status					0.280
New	481	80.03	9.00	4.00–13.00	
Relapse	120	19.97	10.00	5.25–13.75	
Previous hospitalizations					0.073
Yes	486	80.87	10.00	5.00–13.00	
No	115	19.13	9.00	3.00–11.00	
Comorbid illness					0.206
Yes	163	27.12	10.00	5.00–14.00	
No	438	72.88	9.00	4.00–13.00	
Self-assessed severity					**< 0.001**
Severe	70	11.65	12.50	8.00–16.25	
Moderate	160	26.62	10.50	7.00–14.00	
Mild	371	61.73	9.00	3.00–11.00	
Self-assessed cure confidence					**< 0.001**
Strong	442	73.54	9.00	3.00–12.00	
Moderate	112	18.64	10.00	6.25–15.00	
No/minor	47	7.82	11.00	9.00–15.00	
Self-assessed health status					**< 0.001**
Good	275	45.76	9.00	3.00–11.00	
Moderate	262	43.59	10.00	6.00–14.00	
Poor	64	10.65	11.00	7.25–17.00	
Substance (cigarette or alcohol) use					0.210
Yes	142	23.63	9.00	3.00–12.00	
No	459	76.37	10.00	5.00–13.00	

### Correlations between anxiety, social support, doctor-patient communication and TB-related stigma

Among the respondents, the mean anxiety score was 4.03, and the median score was 2. Most of the patients (65.89%) had no or minimal anxiety, while those with moderate and severe anxiety accounted for 6.66 and 5.82% of the sample, respectively. The correlation analyses showed that anxiety was positively correlated with TB-related stigma (*r* = 0.440, *P* < 0.001). Among the participants, the average scores for social support and doctor-patient communication were 25.41 (SD = 5.33) and 17.17 (SD = 2.81), respectively. The correlation analyses found that social support and doctor-patient communication were negatively correlated with TB-related stigma (*r* = − 0.289 and *r* = − 0.233, respectively, *P* < 0.001) (Table 3).

**Table 3:** Anxiety, social support and doctor-patient communication in TB patients

Variables	Median (*P*_25_, *P*_75_)	Mean ± SD	Correlation with TB-related stigma	
			Correlation coefficients	*P*
Anxiety	2.00 (0.00, 7.00)		0.440	**< 0.001**
Social support		25.41 ± 5.33	−0.289	**< 0.001**
Doctor-patient communication		17.17 ± 2.81	−0.233	**< 0.001**

### Patient characteristics associated with TB-related stigma

The multiple linear regression results showed that female patients had higher stigma scores than male patients (*β* = 1.19, 95% *CI*: 0.38–2.01, *P* < 0.05), and patients with moderate and severe conditions had higher stigma scores than patients with mild conditions (*β* = 1.08, 95% *CI*: 0.12–2.03 and *β* = 1.36, 95% *CI*: 0.03–2.70, respectively, *P* < 0.05). In addition, patients with high anxiety scores (*β* = 0.38, 95% *CI*: 0.30–0.46, *P* < 0.001) had high stigma scores. However, patients with good social support (*β* = − 0.25, 95% *CI*: − 0.33--0.17, *P* < 0.001) and doctor-patient communication (*β* = − 0.14, 95% *CI*: − 0.29--0.00, *P* < 0.05) had lower stigma scores. Thus, sex, self-assessed disease severity, anxiety, social support, and doctor-patient communication can be used to predict TB-related stigma (Table 4).

**Table 4:** Multiple linear regression model to determine the factors associated with TB-related stigma

Variables	Estimate	95% CI		SE	*t*	*P*
		Lower	Upper			
Sex (Ref: Male)						
Female	1.19	0.38	2.01	0.42	2.88	**0.004**
Unemployment (Ref: Yes)						
No	−0.26	−1.11	0.59	0.43	−0.59	0.553
Family size (Ref: ≥3)						
1	0.04	−1.61	1.69	0.84	0.05	0.961
2	0.37	−0.62	1.36	0.50	0.73	0.465
Self-assessed severity (Ref: Mild)						
Severe	1.36	0.03	2.70	0.68	2.01	**0.045**
Moderate	1.08	0.12	2.03	0.49	2.22	**0.027**
Self-assessed cure confidence (Ref: No/minor)						
Strong	−0.58	−2.14	0.97	0.79	−0.74	0.461
Moderate	−0.29	−2.00	1.43	0.87	−0.33	0.743
Self-assessed health status (Ref: Poor)						
Good	−0.73	−2.19	0.73	0.75	−0.98	0.327
Moderate	0.02	−1.37	1.41	0.71	0.02	0.982
Anxiety	0.38	0.30	0.46	0.04	9.25	**< 0.001**
Social support	−0.25	− 0.33	− 0.17	0.04	− 6.34	**< 0.001**
Doctor-patient communication	−0.14	− 0.29	− 0.00	0.07	− 1.98	**0.048**
Constant	16.36	12.81	19.91	1.81	9.05	< 0.001

*Ref* reference

## Discussion

In addition to biological, cultural and economic factors, stigma also plays an important role in effective TB control. To the best of our knowledge, this study was the first to investigate stigma and its associated factors among TB patients in Dalian, Northeast China. Among the study participants, the mean score of TB-related stigma was 9.07, which was lower than in a study conducted in Central China [29]. The results showed that 50.42% of the participants reported moderate or high stigma. Of these, 30.95% were classified as having moderate stigma and 19.47% as having high stigma. The results were higher than those from Shandong Province (45.32%) in Eastern China [13]. Compared with other regions, the results were similar to those in studies conducted in Karnataka (51.02%) [6] and India (51.2%) [23], higher than those in Ethiopia (42.4%) [15] and lower than those in Zambia (81.9%) [7]. These variations may be attributed to differences in stigma measurement tools, sample sizes, study areas and participant characteristics. This result revealed that the stigma was high among TB patients in Dalian, Northeast China. In addition, the study also found that 48.42% of the patients were reluctant to tell their friends or colleagues about their illness. This percentage was lower than that in a study of TB patients initially diagnosed in Taiwan, in which 75% of patients reported keeping their TB status a secret [28]. More than half of the patients avoided socializing with others after developing TB, which was similar to percentages reported in previous articles [6, 17].

The World Health Organization (WHO) reported that adult males accounted for the largest proportion of the TB burden [1]. In our study, males also accounted for a large proportion, which was approximately 1.67 times that of female patients. Importantly, our study found that female patients were more likely to have high levels of TB-related stigma. Females tend to have lower social and economic statuses, and when they develop TB, they suffer from social exclusion and discrimination, bear a greater burden regarding health care avoidance, and encounter more stigmatism-related problems than males [5, 7]. Females’ social expectations are mostly associated with family, and TB affects society’s expectations of females to be good mothers and their ability to fulfil the role of wife. This has a disproportionate impact on females, making them more likely to be associated with stigma than males [40, 41]. The negative impact of TB on marriage prospects has been identified as one aspect of TB stigma, and the detrimental impact on a female’s marriage prospect is far greater than that of a male’s [41, 42]. In addition, female patients are generally less educated and have less TB-related knowledge, and lack of TB knowledge has been significantly associated with high TB-related stigma [16, 29]. Studies conducted in other parts of China have not found a correlation between sex and stigma among TB patients [28]. Other sociodemographic characteristics, such as age, education, and marital status, were not shown to be associated with TB-related stigma, similar to results of studies conducted in Swaziland and Central China [29, 43] but different from those in a study conducted by Shivapujimath et al. [6], who found that age and education influenced the stigma of TB patients. That study also revealed that patients who self-assessed their condition as moderate or severe were more likely to have TB-related stigma than those who assessed their condition as mild. This result was similar to that in study that found that the severity of the disease was a good predictor of psychological distress in TB patients [13]. It is understandable that the more severe the illness, the more obvious the symptoms are. The main symptom of TB is chronic cough, and evident TB symptoms can lead to unintended disclosure, increasing levels of anticipated and experienced stigma [9]. These results suggest that particular attention should be paid to female patients and patients with moderate or severe disease when addressing the stigma of TB patients.

The study indicated that anxiety was positively correlated with TB-related stigma. The prevalence of anxiety was significantly higher among patients with perceived TB-related stigma [24]. In addition, stigma also contributed significantly to the level of anxiety in people living with HIV [9]. People who experience TB-related stigma may have a poor self-image and become increasingly isolated, which may lead to increased susceptibility to anxiety disorders [44]. Among the participants, 34.11% reported some degree of anxiety, a proportion lower than those in Pakistan and Brazil [45, 46]. This is a reminder that additional focus should be placed on patients who are likely to develop anxiety. Studies have shown that patients who are female, are in the intensive phase of TB treatment, have comorbid HIV infection, and have poor social support are more likely to have anxiety [24, 47]. Given the strong correlation between stigma and anxiety, an effective set of interventions is urgently needed to reduce the psychological distress caused by the disease.

Social support can be used to address the significant negative consequences of stigma, and it is an important factor in overcoming stigma in patients [33]. The current study also revealed that social support was significantly associated with TB-related stigma. Because people fear being infected, TB patients often experience isolation and separation from their families and communities; some lose their jobs, are shunned by friends and neighbours, have little access to education, and are denied access to shared appliances and food by family members [17, 48]. These negative societal experiences prompt feelings of stigma in patients, causing them to hide their illness and avoid contact and interaction with others. Patients may also internalize these negative attitudes and behaviours, which manifest as shame and low self-esteem. Patients generally report that they want support during their TB illness [49]. Social support for TB patients through TB club activities has been successful in reducing TB-related stigma [50]. These findings demonstrate that providing a variety of social support to TB patients plays a significant role in reducing TB-related stigma. Social support is also important in reducing stigma levels among people living with HIV [51]. Therefore, it is helpful to include social support in TB-related stigma interventions.

Our research found that good doctor-patient communication played an important role in reducing TB-related stigma. Similar results indicating that nurses’ behaviours influenced patients’ perceptions of stigma, with sincerity, empathy and respect as the strongest and most important influencing factors, have been reported [43]. Health care workers are critical in successful TB treatment and management, and their support and care can promote patient motivation and adherence to treatment [52]. However, their negative attitudes and behaviours towards TB patients may lead to greater discrimination against TB patients [53]. There has been plenty of evidence that health professionals may reinforce stigma, particularly in the areas of HIV and mental health. Our study supports evidence that doctor-patient communication is significantly associated with TB-related stigma. At present, there is a general lack of guidance for health workers on building rapport with patients during interviews. Therefore, targeted training for health workers to improve their interactions with patients and to build good doctor-patient communication is important to alleviate stigma in TB patients.

There are several limitations in the current study that must be mentioned. First, this study was a cross-sectional study, limiting the establishment of causal relationships between variables. Therefore, longitudinal studies are needed. Second, we measured TB-related stigma in terms of negative experiences, emotional responses and coping styles among only TB patients and not the public. Third, we conducted only quantitative analyses and not comprehensive assessments of stigma including qualitative analyses. Fourth, we included only patients who had access to medical services and not those who did not. Including these patients may allow a deeper understanding of TB-related stigma. Fifth, other variables, such as self-esteem and family functioning, may also have impacts on stigma, which our study did not address. Finally, this study was carried out in only Dalian and was limited by differences in social and cultural backgrounds among different regions. The results only represent regions with the same conditions and are not generalizable to other regions. Future studies should include an expanded population, enriched investigation methods, additional influencing factors, and different regions.

## Conclusion

This study examined the status of TB-related stigma and its associated factors among TB patients. TB-related stigma was widespread among participants. Patients who were female, had self-assessed moderate or severe disease, and had anxiety were more likely to have a high level of TB-related stigma than their counterparts. However, patients with good social support and doctor-patient communication were more likely to stay away from TB-related stigma. Therefore, when conducting TB stigma-related interventions, more resources should be allocated to female patients and patients with moderate or severe disease. Moreover, the important role of social support and doctor-patient communication in reducing TB-related stigma should also be emphasized.

## Supplementary Information

**Additional file 1.** Questionnaire to assess tuberculosis-related stigma among tuberculosis patients in Dalian. The questionnaire collected information about sociodemographic characteristics, TB-related stigma, treatment and substance use statuses, anxiety status, social support, and doctor-patient communication.

## Data Availability

The datasets used and/or analysed during the current study are available from the corresponding author upon reasonable request.

## Abbreviations

TB: Tuberculosis
HIV: Human immunodeficiency virus
AIDS: Acquired immune deficiency syndrome
GDP: Gross domestic product
GAD-7: 7-item Generalized Anxiety Disorder scale
SSRS: Social Support Rating Scale
WHO: World Health Organization
SD: Standard deviation
CI: Confidence interval
SE: Standard error
