# Supplementary material for: Tuberculosis-related stigma and its determinants in Dalian, Northeast China: a cross-sectional study
Source: BMC Public Health. 2021 Jan 4;21:6. doi: 10.1186/s12889-020-10055-2 (PMC7780403; doi:10.1186/s12889-020-10055-2)
Supplement: Supplementary file 1 — Additional file 1. Questionnaire to assess tuberculosis-related stigma among tuberculosis patients in Dalian. The questionnaire collected information about sociodemographic characteristics, TB-related stigma, treatment and substance use statuses, anxiety status, social support, and doctor-patient communication. [file 12889_2020_10055_MOESM1_ESM.pdf]

Questionnaire to assess tuberculosis-related stigma among tuberculosis patients in Dalian

Hello! We are the tuberculosis (TB) research team at Dalian Medical University, and we are conducting a survey on TB-related stigma. The aim of this survey was to assess TB-related stigma and its determinants among TB patients in Dalian. The data collected will only be used for scientific research and will not have any adverse impact on you personally. We are asking you for your generous help. We assure you that the content of your answers will be kept strictly confidential. If you do not want to participate in the study, you have full right to refuse at any time. But your honest participation will have a great contribution. So please take a few minutes to answer these questions. Thank you for your cooperation!

Part I: Socio-demographic characteristics

No	Questions	Coding Categories	Code
101	Sex	1. Male 2. Female	
102	Age	________years old	
103	Employment status	1. Employment 2. Unemployment	
104	Marital status	1. Unmarried<br>2. Married<br>3. Divorced or widowed	
105	What is your highest completed level of education?	1. Primary or less<br>2. Secondary<br>3. High school or above	
106	How many people live together in your family?	1. 1<br>2. 2<br>3. ≥3	

Part II: TB-related stigma

Below is a list of the ways you might have felt or behaved. Please tell me how much you feel this way since you have TB.

0 = Strongly disagree 1 = Disagree 2 = Agree 3 = Strongly agree

No	Questions	Coding Categories				Code
201	I feel ashamed of myself for having TB.	0	1	2	3	
202	I am unwilling to reveal my TB to my friends or neighbors.	0	1	2	3	
203	I think less of myself for having TB.	0	1	2	3	
204	I feel that my family members look down on me for having TB.	0	1	2	3	
205	I feel that my friends or neighbors look down on me for having TB.	0	1	2	3	
206	I feel that my friends or neighbors look down on my	0	1	2	3	

	family because I have TB.					
207	I feel that my friends or neighbors avoid me on purpose.	0	1	2	3	
208	I avoid keeping in touch with my friends or neighbors since I have TB.	0	1	2	3	
209	I am afraid of going to TB clinics because other people may see me there.	0	1	2	3	

Part III: Condition of treatment and substance use

No	Questions	Coding Categories	Code
301	TB status	1. New 2. Relapse	
302	Have you ever been hospitalized for TB?	1. Yes 2. No	
303	Do you have comorbidity?	1. Yes 2. No	
304	Do you think your condition is serious?	1. Serious<br>2. Moderate<br>3. Mild	
305	Are you confident of a cure for TB?	1. Strong<br>2. Moderate<br>3. No/minor	
306	What do you think of your current general status of health?	1. Good<br>2. Moderate<br>3. Poor	
307	Are you smoking now?	1. Yes 2. No	
308	Are you drinking alcohol now?	1. Yes 2. No	

Part IV: Anxiety status

Over the last 2 weeks, how often have you been bothered by the following problems?

0 = Not at all 1 = Several days 2 = More than half the days 3 = Nearly every day

No	Questions	Coding Categories				Code
401	Feeling nervous, anxious or on edge	0	1	2	3	
402	Not being able to stop or control worrying	0	1	2	3	
403	Worrying too much about different things	0	1	2	3	
404	Trouble relaxing	0	1	2	3	
405	Being so restless that it is hard to sit still	0	1	2	3	
406	Becoming easily annoyed or irritable	0	1	2	3	
407	Feeling afraid as if something awful might happen	0	1	2	3	

Part V: Social support

501. How many close friends do you have who can provide support and help?

1. None 2. 1 or 2 3. 3 to 5 4. 6 or more

502. How has your residence been in the past year?

1. Live alone in one room, away from the family
2. Living quarters change frequently and spend most of the time with strangers

3. Live with classmates, colleagues or friends
4. Live with family

503. How do you get along with your neighbors?
1. Never care about each other
2. Might be slightly concerned when in trouble
3. Some neighbors are concerned about you
4. Most of your neighbors care a lot about you

504. How do you relate to your (current or former) colleagues?
1. Never care about each other
2. Might be slightly concerned when in trouble
3. Some colleagues are concerned about you
4. Most of your n colleagues care a lot about you

505. How much support and care do you receive from family members? (Put "√" in the appropriate box)

	Nothing	Few	General	Full support
Husband or wife (lovers)				
Parents				
Sons and daughters				
Brothers and sisters				
Other members (e.g. sister-in-law)				

506. Where do you get financial help or solutions when you are in trouble? (Multiple choice)
1. Spouse 2. Other family members 3. Relatives 4. Colleagues 5. Work units
6. An official or semi-official organization such as a party, league, or trade union
7. Non-official organizations such as religious and social organizations
8. Other____________ 9. Without any source

507. Where do you get comfort and care when you are in trouble? (Multiple choice)
1. Spouse 2. Other family members 3. Relatives 4. Colleagues 5. Work units
6. An official or semi-official organization such as a party, league, or trade union
7. Non-official organizations such as religious and social organizations
8. Other____________ 9. Without any source

508. What is the way you talk about your troubles?
1. Never confided in anyone
2. Only confide in one or two people who are very close to you
3. If a friend asks, you speak up
4. Offer to talk about your troubles to get help

509. What is the way to ask for help when you are in trouble?
1. Rely on yourself and never accept help from others
2. Seldom ask for help
3. Sometimes ask for help
4. Often ask for help from family, friends and organizations when in trouble

510. How often will you participate in the activities of organizations (social) such as party organizations, religious organizations, trade unions, etc.?
1. Never 2. Occasionally 3. Sometimes 4. Often

5. Be initiative to participate and be active

Part VI: Doctor-patient communication

Please choose the answer that you think best suits you.

1 = Very satisfied 2 = Satisfied 3 = Relatively satisfied 4 = Dissatisfied 5 = Very dissatisfied

No	Questions	Coding Categories					Code
601	How satisfied are you with your doctor's explanation of your condition?	1	2	3	4	5	
602	How satisfied are you with your doctor's explanation of the usage and dosage of anti-TB drugs?	1	2	3	4	5	
603	How satisfied are you with your doctor's explanation of adverse reactions to anti-TB drugs?	1	2	3	4	5	
604	In the course of TB treatment, how satisfied are you with the doctor's service attitude?	1	2	3	4	5	

Thank you again for participating in this survey! I wish you a speedy recovery!

Investigate member:__________(signature) The reviewer:__________(signature)

Survey date:_________________
